# Identification of a type I Ca^2+^/Mg^2+^-dependent endonuclease induced in maize cells exposed to camptothecin

**DOI:** 10.1186/1471-2229-13-186

**Published:** 2013-11-20

**Authors:** Núria Sánchez-Pons, Carlos M Vicient

**Affiliations:** 1 Department of Molecular Genetics, Centre for Research in Agricultural Genomics CRAG (CSIC-IRTA-UAB-UB), Campus UAB, Bellaterra (Cerdanyola del Vallès), 08193 Barcelona, Spain

**Keywords:** Camptothecin, DNA degradation, Maize, Nuclease

## Abstract

**Background:**

Camptothecin is a plant alkaloid that specifically binds topoisomerase I, inhibiting its activity and inducing double stranded breaks in DNA and activating the cell responses to DNA damage.

**Results:**

Maize cultured cells were incubated in the presence of different concentrations of camptothecin. Camptothecin inhibits cultured cell growth, induces genomic DNA degradation, and induces a 32 kDa Ca^2+^/Mg^2+^-dependent nuclease activity. This nuclease, we called CaMNUC32, is inhibited by Zn^2+^ and by acid pH, it is mainly localized in the nucleus and it cleaves single- and double-stranded DNA, with a higher activity against single-stranded DNA. Two-dimensional electrophoresis combined with mass spectrometry suggests that CaMNUC32 is a member of the type I S1/P1 nuclease family. This type of nucleases are usually Zn^2+^-dependent but our results support previous indications that S1-type nucleases have a wide variety of enzyme activities, including Ca^2+^/Mg^2+^-dependent.

**Conclusions:**

We have identified and characterized CaMNUC32, a 32 kDa Ca^2+^/Mg^2+^-dependent nuclease of the S1/P1 family induced by the topoisomerase I inhibitor camptothecin in maize cultured cells.

## Background

Topoisomerase I (TOPI) is an enzyme which catalyses the relaxation of super coiled DNA by cleaving and re-joining one DNA strand [1]. TOPI activity is essential in dividing cells to release the torsion created by the progression of DNA replication forks. Camptothecin (CPT) is a plant alkaloid that specifically binds to TOPI, stabilizing the complexes formed between DNA and TOPI [2]. The collisions between the trapped TOPI-CPT complexes and the replication fork during DNA replication produce DNA breaks, which induce DNA damage responses and, depending on the dose, cell death [3]. CPT-induced DNA damage responses have also been observed in plants [4]. For example, CPT induces DNA damage in maize immature embryos and induces the transcription of genes involved in DNA repair, the repression of genes involved in cell division, the accumulation of several proteins involved in stress responses and the induction of the activity of some nucleases [5]. Nuclease activation in response to DNA damaging agents has been previously observed [6-8].

Eukaryotic nucleases are involved in several processes like DNA restriction, repair, recombination, transposition and programmed cell death (PCD) [9-11]. Plant nucleases can be classified into groups depending on their catalytic properties, mainly their divalent metal ion requirements. Plants have two major classes of endonucleases, Zn^2+^-dependent and Ca^2+^-dependent endonucleases [9,12] and refereed articles. Some other subgroups have been identified whose nucleolytic activities are stimulated by other ions such as Mg^2+^[13], Mn^2+^[14] or Co^2+^[15].

Zn^2+^-dependent nucleases (also termed Type I nuclease or S1-type nucleases) includes acidic and neutral enzymes with a molecular mass between 30 and 45 kDa and that efficiently degrade RNA and denatured DNA, but not double stranded DNA. A number of Zn^2+^-dependent nucleases have been described in plants as, for example, the mung bean nuclease [16], ZEN1 from *Zinnia elegans* involved in tracheary element differentiation [17], Arabidopsis BFN1 [18] and ENDO2 [19], and others [20]. Ca^2+^-dependent nucleases include neutral enzymes [8,17,21]. For example, Arabidopsis Ca^2+^-dependent CAN nuclease [22], *Eucommia ulmoides* Oliv. EuCaN1 and EuCaN2 Ca^2+^-dependent nucleases involved in the secondary xylem development [23], and cucumber Ca^2+^-dependent nuclease CsCaN involved, among other possible functions, in the primordial anther-specific DNA damage of developing female cucumber flowers [24].

Determination of the catalytic requirements of a nuclease is essential for understanding its biological function. In normal conditions, plant cell nucleus and cytoplasm have a neutral pH (around 7.5) and low concentrations of Ca^2+^ and Zn^2+^, and the apoplast and vacuoles have a more acidic pH (around 5.5) [25]. Vacuoles usually have a higher concentration of Zn^2+^, and apoplast a higher concentration of Ca^2+^ than the cytoplasm. However, this situation can change in response to different stimulus. For example, during PCD the tonoplast rupture produces a higher concentration of Zn^2+^ in the cytosol which also becomes more acid [26]. It has also been reported that some stresses rise cytosolic concentration of calcium due to an increase in the Ca^2+^ influx from apoplasts [27]. These changes may alter the activity of particular nucleases.

In this paper, we identify and characterize a Ca^2+^/Mg^2+^-dependent nuclease whose activity is induced by CPT in maize cultured cells. We incubated maize cells with CPT and we observed an increase in a Ca^2+^-dependent nuclease activity similar to what it was previously observed in maize CPT-treated embryos [5]. This nuclease has a mass of 32 kDa, is activated by Ca^2+^ and Mg^2+^, and inhibited by Zn^2+^ and EDTA. It cleaves either single- and double-stranded DNA, with a higher activity against single-stranded DNA and is mainly localized in the nucleus. Two-dimensional in-gel assays and MALDI-TOF MS approaches following in-gel tryptic digestion allowed us to identify the protein responsible of the nuclease activity, which belongs to the S1/P1 type I endonuclease family.

## Results

### Induction of growth arrest and DNA fragmentation by camptothecin in maize cultured cells

**Figure 1:**
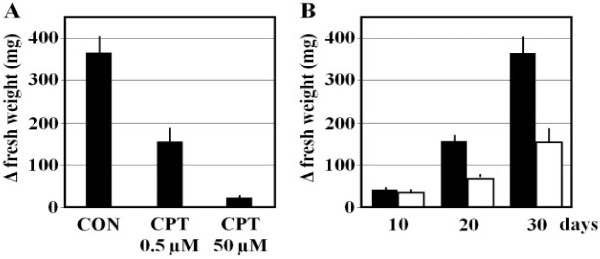
**The effect of camptothecin (CPT) on the growth of maize cultured cells. (A)** An initially similar number of maize cells (3.3 ± 0.5 mg) were grown on MS medium supplemented with DMSO (Control, CON) or with different concentrations of CPT during 30 days. The difference in fresh weight between day 0 and day 30 is shown. **(B)** Maize cells were grown on MS medium supplemented with DMSO (black bars) or with 0.5 μM CPT (white bars) throughout different number of days. The difference in fresh weight between day 0 and the indicated day is represented. The graphics depict the average of three independent experiments, and error bars (SE) are annotated.

The topoisomerase I inhibitor camptothecin (CPT) produces a reduction of maize cultured cell growth when added to the culture medium (Figure 1A). A concentration of 0.5 μM CPT reduces the increase in fresh weight of callus to 58% compared to control and 50 μM CPT produces a reduction in 94% of the increase in fresh weight. Growth inhibition is persistent, so the differences in the growth of fresh weight increased with time (Figure 1B).

### A nuclear-localized Ca^2+^/Mg^2+^-dependent endonucleolytic activity induced by CPT in cultured cells

**Figure 2:**
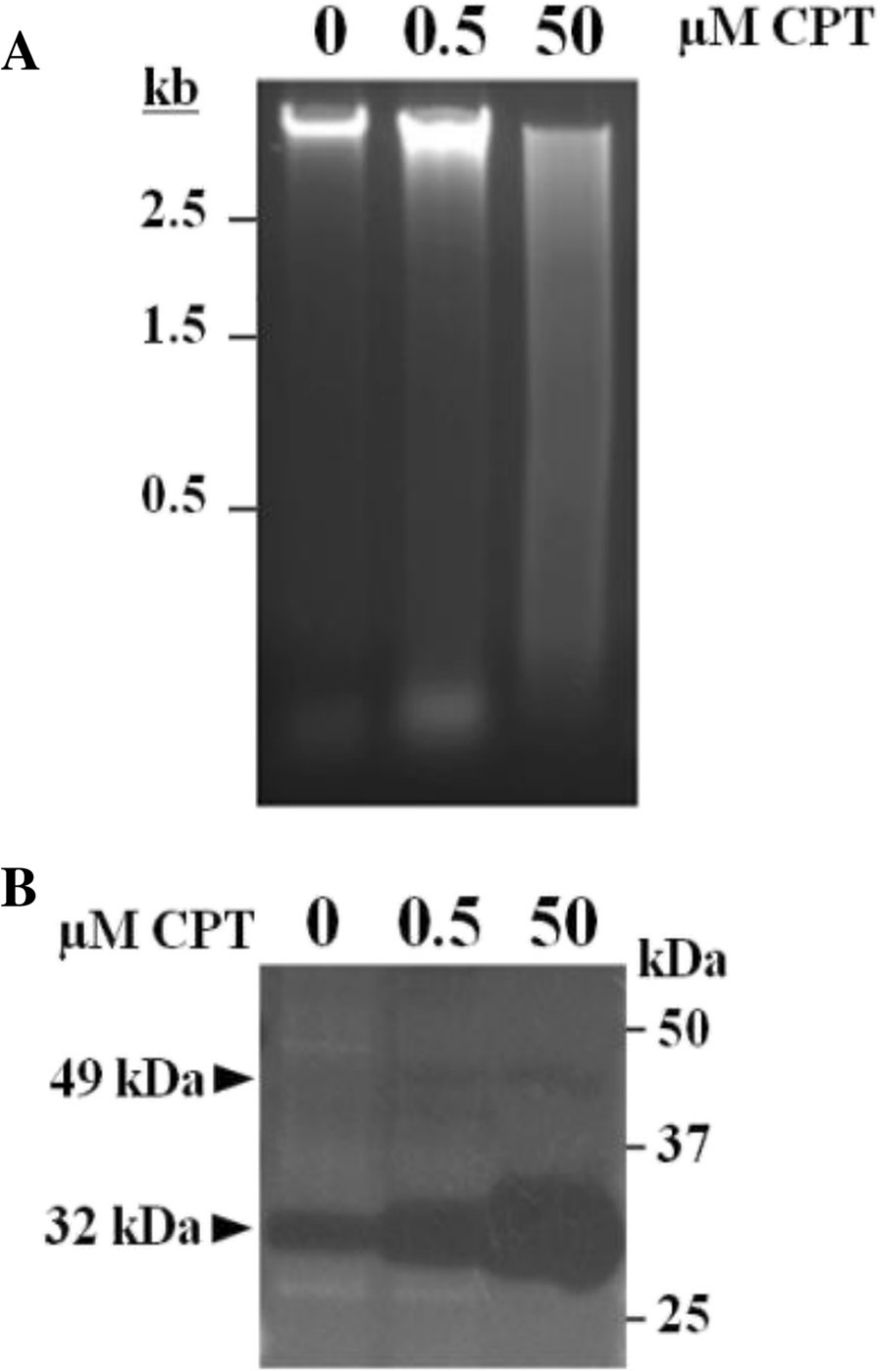
**Biochemical and molecular characterization of the CPT effect on maize cultured cells. (A)** Pattern of DNA fragmentation of maize cultured cells in response to CPT treatments. Maize cells were grown on MS medium supplemented with DMSO (control, 0) or with different concentrations of CPT during 30 days. Afterwards, the cells were frozen in liquid nitrogen and the genomic DNA isolated. The same amount of genomic DNA (4 μg) from each sample was fractionated on 1.5% agarose gel. The sizes of the DNA molecular markers are indicated (kb). **(B)** Identification of a CPT-induced nuclease in maize cultured cells. Nuclease activity assays were performed with 15 μg of total protein extracted from maize cultured cells incubated with different concentrations of CPT during 30 days. The assays were carried out in the presence of 1 mM CaCl_2_ and 1 mM MgCl_2_ in 10 mM Tris–HCl pH 7.5.

The average size of the genomic DNA fragments extracted from CPT-treated maize cultured cells are significantly shorter than the extracted from untreated cells (Figure 2A). These differences are especially evident in cells treated with 50 μM CPT, but are also apparent in the case of cells treated with 0.5 μM CPT.

The nucleases present in the cultured cells were analyzed by in-gel DNase activity assays in the presence of Ca^2+^ and Mg^2+^ (Figure 2B). An endonuclease activity with a molecular mass of 32 kDa was detected in protein extracts of treated and untreated cells, but the activity level was positively correlated with the concentration of CPT in the media. A second nuclease activity with an estimated molecular mass of 49 kDa was also observed, although its activity was much lower and did not change in response to CPT.

**Figure 3:**
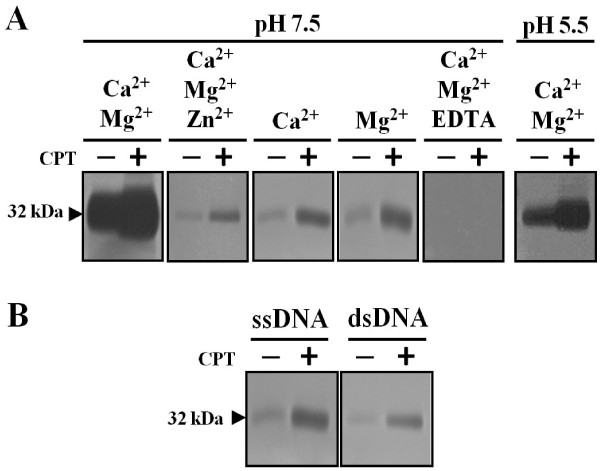
**Characterization of CaMNUC32 nuclease activity. (A)** Protein extracted from maize cultured cells (15 μg) treated with 0.5 μM CPT (+) or untreated (-) during 30 days were subjected to in-gel DNase activity assay under different conditions of pH and cationic requirements. The conditions of the nuclease assays are indicated: Ca^2+^, 1 mM CaCl_2_; Mg^2+^, 1 mM MgCl_2_; Zn^2+^, 5 mM ZnSO_4_; EDTA, 1 mM EDTA. **(B)** Nuclease activity assays were performed in presence of calf thymus single stranded DNA (ssDNA) or salmon sperm double stranded DNA (dsDNA), in 1 mM CaCl_2_, 1 mM MgCl_2_ and pH 7.5.

In order to determine the catalytic requirements of the 32 kDa CPT-induced nuclease we used in-gel nuclease assays under different incubation conditions (Figure 3A). We analyzed the influence on nuclease activity of three divalent metal ions: Zn^2+^, Ca^2+^ and Mg^2+^. Zn^2+^ and Ca^2+^ are the most common metal ion cofactors of plant nucleases and much less is known about the role of Mg^2+^ on nuclease activity. The 32 kDa nuclease activity is only slightly present in the presence of Ca^2+^ or in the presence of Mg^2+^, although in both cases is higher in the CPT-treated samples compared to the control. However, a very much higher activity was obtained when the gels were incubated in the presence of both, Ca^2+^ and Mg^2+^, showing an increased activity in CPT-treated cells. The Ca^2+^ and Mg^2+^-dependent nucleolytic activity was strongly inhibited by 1 mM EDTA and, although not completely, by Zn^2+^, at least in our conditions. This finding confirms the common observation that plant Ca^2+^-dependent nuclease activity can be inhibited by Zn^2+^[9]. Due to the mass and cationic preferences, we called this nuclease CaMNUC32 (Ca^2+^/Mg^2+^-dependent Nuclease of 32 kDa).

pH is another important factor determining nuclease activity. The majority of the plant nucleases have the highest activity at acidic or at neutral pH [21]. We compared the intensity of the 32 kDa nuclease activity in the presence of Ca^2+^ and Mg^2+^ incubated at pH 5.5 and pH 7.5 (Figure 3A). Although the activity was also observed at pH 5.5, it was much lower than at pH 7.5. In both cases, the activity was higher in the samples extracted from CPT-treated cells.

Plant nucleases show divergent specificities towards their substrates, so, we analyzed the nucleolytic activity using single-stranded and double-stranded DNA. The 32 kDa nuclease is able to hydrolyze both single and double stranded DNA, but shows higher activity against single stranded molecules (Figure 3B). The higher affinity for single stranded DNA did not change in samples treated or not with CPT.

### CaMNUC32 subcellular localization and isoforms

**Figure 4:**
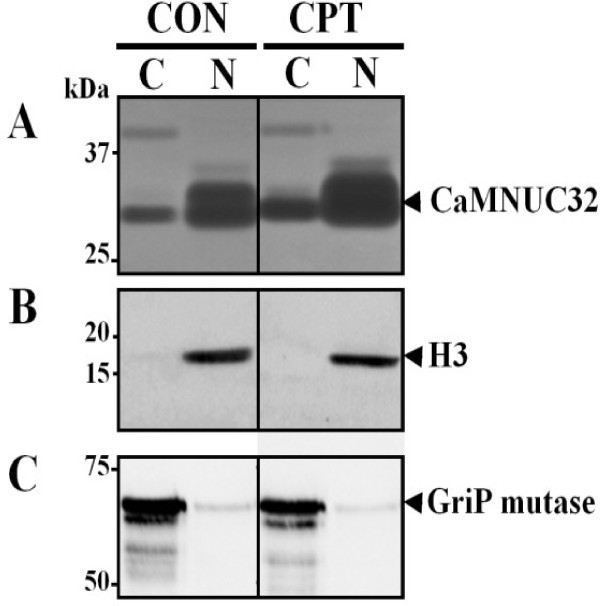
**Subcellular localization of CaMNUC32 nuclease activity.** Maize cell cultures were grown in the absence or presence of 0.5 μM CPT during 20 days, and fractionated into nuclear and cytoplasmic fractions. Equal among of protein from cytoplasmic and nuclear extracts were subjected in parallel to in-gel activity assay and Western-blot analysis. **(A)** In-gel Ca^2+^/Mg^2+^-dependent nuclease activity assay of cytoplasmic and nuclear fractions. Nuclear fraction purity was checked by immunological detection of the nuclear protein Histone-3 **(B)** and the cytoplasmic enzyme GriP mutase **(C)**. CON, non-treated ells; CPT, cells treated with 0.5 μM CPT; C, cytoplasmic extract; N, nuclear extract.

In order to determine the subcellular localization of CaMNUC32 we performed cellular fragmentation experiments. Nuclease activities were analyzed in cytoplasm and nucleus extracts (Figure 4A). Nuclear fraction purity was checked by immunological detection of a nuclear localized protein (Histone 3, Figure 4B) and a cytoplasmic localized protein (GriP mutase, Figure 4C). At least four nuclease activity bands were observed in these assays. The previously observed (Figure 2B) approximately 49 kDa nuclease activity which is not induced in CPT-treated samples was only present in the cytoplasm. The nucleolytic activity of CaMNUC32 was mostly, but not exclusively, localized in the nuclear fraction, localization that did not change in the CPT-treated samples.

**Figure 5:**
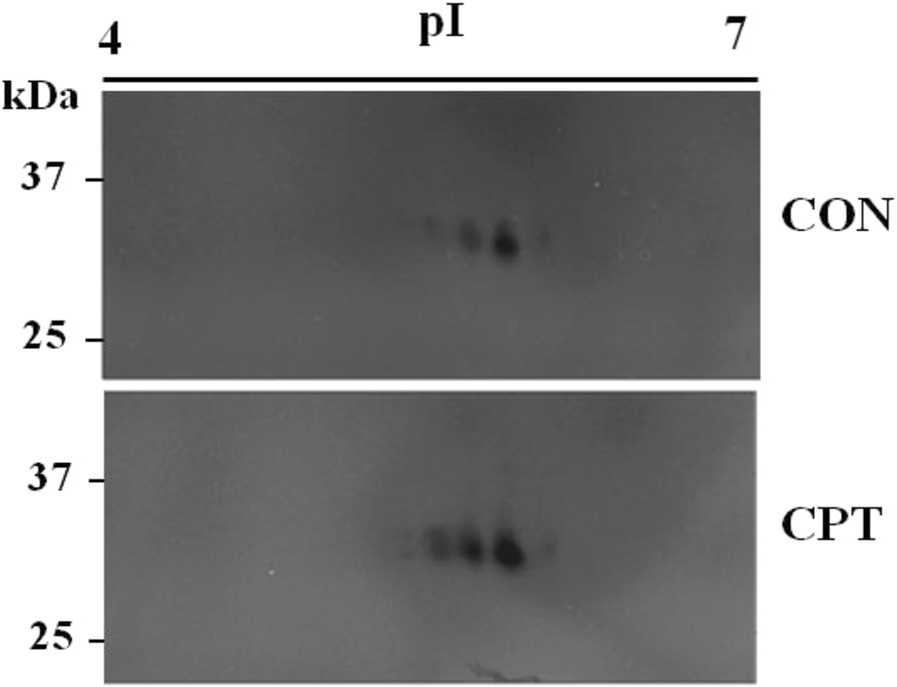
**Identification of CaMNUC32 isoforms by two-dimensional in-gel nuclease activity assay.** Nuclease activities of total protein extracts (125 μg) subjected to 2D-nuclease activity assays (IEF in 12 cm strips). (CON) Total protein extract from cultured cells treated with control medium supplemented with DMSO during 30 days. (CPT) Total protein extract from cultured cells treated with 0.5 μM CPT during 30 days (CPT).

Two bands of nuclease activity were observed in these in-gel assays with a molecular weight very close to CaMNUC32 (Figure 4A). Both nuclease activities are observed in the nuclear samples, and the intensities of both bands are higher in the CPT-treated samples. The similar behavior against CPT, the similar subcellular localization and the similar size suggested that they are different isoforms of the same nuclease, although we cannot also discard the possibility that they correspond to different nucleases. In order to clarify this point, we attempted 2D-in gel nuclease activity assays. First, we examined if CaMNUC32 activity was conserved in the conditions used for 2D gels. The presence of the alkylating agent iodoacetamide (IAA) strongly inhibits CaMNUC32 nuclease activity, whereas the reducing agent dithiothreitol (DTT) reduces its activity only slightly (Additional file 1: Figure S1). Subsequently, the 2D experiments were carried out using a standard protocol but omitting the second strip equilibration step that uses IAA. When total extracts were subjected to 2D gel analysis, the CaMNUC32 activity was divided in four main spots with minor differences in molecular weight but showing different isoelectric properties (pI 5.5, pI 5.7, pI 6.0, pI 6.2, approximately) (Figure 5). CPT produces the increase in the intensity of all four spots, besides an additional activity located around pI 5.3, but did not produce a significant change in the isoelectric properties of CaMNUC32. The pattern of distribution of the nuclease activities suggests that CaMNUC32 protein suffers post-translational modifications, but we cannot rule that they correspond to different nucleases with similar mass and different pI.

### CaMNUC32 is a type I endonuclease of the S1/P1 family

**Figure 6:**
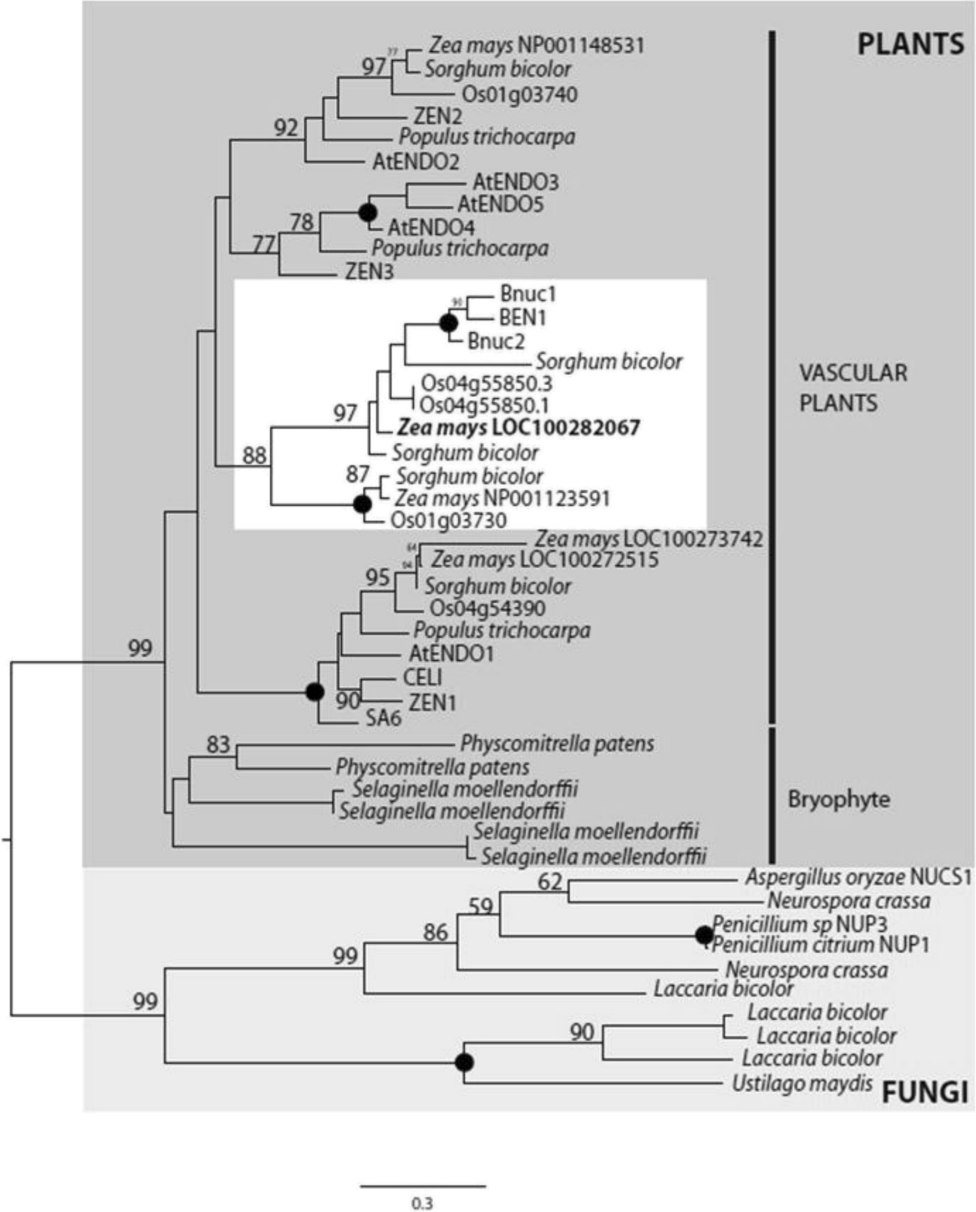
**Phylogenetic analysis of type I nucleases.** Putative protein sequences of plant species with complete genome sequenced available were obtained from JGI Genome Portal (http://genome.jgi-psf.org/). The phylogenetic tree was inferred by maximum-likelihood method (RaxML) using 100-bootstraps repeats. For each clade, the significant bootstrap nodal support values (higher than 50%) are annotated, while 100% values are represented by black dots. The maize nuclease identified by mass spectrometry (in bold) locates into a specific group composed by only cereal nucleases (white square), clearly different from those represented by ZEN1/AtENDO1 (group 1) and ZEN2/AtENDO2 (group 2), which include other maize members.

To address the identification of the protein responsible of CaMNUC32 nuclease activity, nuclear protein extracts of maize cells treated with CPT were subjected to 2-DE followed by nuclease activity assay. As previously, four activities were detected with similar mass and different pI (Additional file 2: Figure S2). The three spots with higher activity were excised (H1, H2 and H3). We took only the lower part of the spots in order to avoid the presence of the detected nuclease activity with a slightly higher molecular weight than CaMNUC32 (Figure 4A). After excision of the spots, in-gel trypsin digestion, MALDI-TOF-MS of the tryptic peptides and database searching only H2 spot provided positive data. The digestion of the H2 spot contained one peptide (K.TCADKYAAESAK.L), which matches to a maize PA3 nuclease (GeneBank, NP_001148452), with a Mascot Score of 47 and a protein coverage of 4%. Even though the maize genome survey reveals four putative loci coding for PA3 nucleases, the protein sequence alignment reflects that the tryptic peptide sequence exclusively matches with the maize protein code by gene LOC100282067 (Additional file 3: Figure S3). Interestingly, the predicted mass and pI of this protein is very similar to the observed for H2 spot: 32.0 versus 32.8 kDa, and 5.50 versus 5.55 pI (expected versus observed). The phylogenetic analysis of plant S1-nucleases indicates that three main groups are differentiated in vascular plants, represented by ZEN1/AtENDO1, ZEN2/AtENDO2 and ZEN3/AtENDO3, whilst CaMNCU32 locates into a specific group composed by only cereal nucleases, clearly different from group-1 and group-2 (Figure 6). Thus, maize possesses clear orthologs to group 1, represented by AtENDO1/ZEN1, and group 2 corresponding to AtENDO2/ZEN2 [28]. Due to the low statistical support as well as that any cereal contains clear orthologs into group 3 (AtENDO3/ZEN3), this particular cluster of nucleases is likely to be the group 3 in cereals.

## Discussion

The aim of this study was to identify possible nucleases associated with the camptothecin inhibition of cell growth in maize cultured cells. Our results revealed the existence of at least one nuclease whose activity is induced in parallel to CPT treatment. Our in gel nuclease assays demonstrated that the activity of a 32 kDa nuclease increases during CPT treatment. This activity is higher in neutral compared to acidic pH and is higher in the presence of both Ca^2+^ and Mg^2+^, whereas is reduced in the presence of the single ions. The activity is inhibited by EDTA and reduced by Zn^2+^. Higher nucleolytic activity was observed against ssDNA, although it is also active towards dsDNA. The nuclease activity is mainly, although not exclusively, located in the nucleus.

Bidimensional pattern of nuclease activity exhibited by CaMNUC32 suggests that this protein is subjected to post-translational modifications as it has been reported for some Ca^2+^-dependent nucleases [29], although we cannot discard that different nucleases with similar activity, size and pI exist. Heterologous expression of S1-nucleases in prokaryote systems demonstrated that post-translational events are essential for their activity [28,30,31]. Glycosylation is a good candidate to be responsible for the post-translational changes since the S1-nucleases and Ca^2+^-dependent DNases are considered to be glycoproteins [10,29]. Nevertheless, other post-translational processes might be considered [32].

Ca^2+^/Mg^2+^-dependent nucleases with similar properties have been previously described in different systems. For example, rice OsCyt20 is a Ca^2+^/Mg^2+^-dependent nuclease that shows a higher activity at neutral pH and is inhibited by Zn^2+^[10]. Ca^2+^/Mg^2+^-dependent nuclease has also been identified in wheat grain nucellar cells undergoing PCD and is inhibited by Zn^2+^[13]. A similar nuclease of 28 kDa was observed in calf thymus chromatin [33]. Arabidopsis CAN1 and CAN2 nucleases are neutral, Ca^2+^-dependent nucleases showing a different specificity toward the ssDNA, dsDNA and RNA substrates [22]. However, the identity of the majority Ca^2+^/Mg^2+^-dependent nucleases remains uncertain [8].

Our 2-DE nuclease activity assay associated with spot excision and MALDI-TOF MS following in-gel tryptic digestion suggested that CaMNUC32 is a member of the S1/P1 family of type I endonucleases. Although the results provide a very limited sequence coverage (Additional file 3: Figure S3), the fact that the identified protein is a putative nuclease with a predicted molecular mass and a pI very similar to the observed ones provides enough confidence in the results. Type I S1/P1-nucleases exhibit amino acid sequence homology with nuclease S1 from *Aspergillus oryzae* and are classically defined by requiring Zn^2+^ for activation and structure stabilization, to have a pH optimum in the acidic region, to have between 30 and 43 kDa, and to be able to degrade single-stranded DNA [34,35] although some of them have the capacity to digest both single-stranded and double-stranded DNA [17]. Our results revealed that some S1-type nucleases are Ca^2+^/Mg^2+^dependent nucleases with neutral pH optimum. In fact, this is not the first report on a similar case. For example, the S1-like nuclease CEL1 isolated from celery was reported as a Zn^2+^- and Mg^2+^-dependent enzyme [36], and, recently, it has been shown that the S1-like family of nucleases in Arabidopsis, in addition to Zn^2+^-dependent enzymes, it also comprises nucleases activated by Ca^2+^ and Mn^2+^ and that they differ in their optimum pH value and substrate specificity [12]. Altogether, these results reveal that plant S1-like nucleases have a surprisingly wide variety of enzyme activities. It also demonstrates that the biochemical classification of the plant nucleases does not perfectly fit their phylogenetic classification. Type I S1/P1-nuclease cannot be considered anymore as synonymous of Zn^2+^-dependent nuclease. The discovery of a wide variety of ions activating or inhibiting plant S1-like nucleases opens new perspectives for future research into reaction mechanisms catalyzed by these enzymes. S1-like family seems to evolve towards increasing the diversity of their catalytic properties. In fungi S1 and P1 nucleases play a role in the degradation of the extracellular DNA and RNA [37,38], whereas in plants, most of the data suggest a relationship between S1-like nucleases and PCD. Further analyses are necessary to demonstrate that CaMNUC32 is involved in PCD. The correlation of its activity with DNA degradation suggests this function. Its mainly nuclear localization is also compatible with a role in PCD, although it is also compatible with other functions like DNA repair or recombination. CaMNUC32 activity, although at a lower extend, can be also observed in the cytoplasm, but this is not inconsistent with a PCD function. For example, the BFN1 nuclease is located in cytoplasmatic structures in early-senescent leaf cells and, as the tissue senesced, BFN1 is observed more abundantly around the nuclei [39]. On the other hand, we also observed CaMNUC32 activity in untreated cells that may be, in principle, contradictory with a PCD function. We cannot discard that some of the cells in the culture are suffering a senescence-like process. On the other hand, nuclease activities have also been detected in tissues where PCD does not occur as, for example, in Arabidopsis mature stems which show low levels of BFN1 activity [18]. Further analyses will be necessary to unequivocally associate CaMNUC32 with PCD.

## Conclusions

We identified a 32 kDa nuclease, that we called CaMNUC32, whose activity is induced by the topoisomerase I inhibitor camptothecin in maize cultured cells. The activity of this nuclease is Ca^2+^/Mg^2+^-dependent and is inhibited by Zn^2+^ and EDTA. It cleaves either single- and double-stranded DNA. MALDI-TOF MS following in-gel tryptic digestion suggests that CaMNUC32 belongs to the S1/P1 type I endonuclease family.

## Methods

### Cell culture and treatments

We used the maize (*Zea mays*) cv Black Mexican Sweet (BMS) as a source of cultured cells. Maize cells were grown on MSE medium (MS vitamins, 20 g l^-1^ sucrose, 0.5 g l^-1^ MES, 2.4-D 2 mg ml^-1^, 2 g l^-1^ gelrite pH 5.8) and maintained at 26°C, 45% humidity and photoperiod 16/8 h. When required, the incubation medium was supplemented with camptothecin (CPT) diluted in DMSO (Sigma) and the same volume of DMSO was added to control experiments. At least three independent experiments were performed per treatment.

### Isolation of DNA and agarose electrophoresis

Maize callus were frozen in liquid nitrogen and ground with a mortar and pestle. DNA was extracted with extraction buffer (100 mM Tris–HCl, pH 8.0, 50 mM EDTA, 500 mM NaCl, 10 mM β-mercaptoethanol, 2% (w/v) SDS). Contaminated RNA was removed by incubation for 10 minutes at 37°C in the presence of RNaseA (60 ng μl^-1^). After extraction with phenol:chloroform:isoamyl alcohol (25:24:1), DNA was precipitated with two volumes of absolute ethanol and resuspended in TE (10 mM Tris–HCl, 1 mM EDTA, pH 8.0) buffer. DNA was resolved on 1.5% (w/v) agarose gels stained with ethidium bromide.

### Preparation of protein extracts

Total protein extracts were obtained from maize callus frozen in liquid nitrogen, ground with a mortar and pestle and resuspended in extraction buffer (150 mM Tris–HCl, pH 6.8, 0.5 mM PMSF, 20 μM leupeptin). The homogenate was clarified by centrifugation at 12.000x g for 5 min at 4°C. Preparation of nuclear and cytoplasmic protein extracts from maize cultured cells was done according to [40].

### In-gel nuclease activity assay

The nuclease activity assays were carried out according to a method previously described [13,41] with minor modifications. Protein extracts (10 μg of protein) were subjected to SDS-PAGE gels containing 50 μg ml^-1^ of single-stranded calf thymus DNA and 50 μg ml^-1^ bovine fibrinogen (Sigma). Samples were prepared in loading buffer (1% (w/v) SDS, 62.5 mM Tris–HCl, pH 6.8, 10% (v/v) glycerol, 10 mM β-mercaptoethanol) and were heated 2 min at 100°C. Electrophoresis was carried out at 20 mA and at room temperature. After electrophoresis, the gels were washed twice for 30 min in 25% (v/v) isopropanol, 10 mM Tris–HCl, pH 7.0 and then twice for 30 min in 10 mM Tris–HCl, pH 7.5. The gels were then incubated overnight at 37°C in 10 mM Tris–HCl, pH 7.5 with some of these compounds: 1 mM CaCl_2_, 1 mM MgCl_2_, 5 mM ZnSO_4_ and/or 1 mM EDTA. pH5.5 assays were performed in the same conditions but replacing 10 mM Tris–HCl pH 7.5 by 25 mM sodium acetate pH 5.5. Finally, the gels were stained with 1 μg ml^-1^ ethidium bromide for 15 min. Nuclease activity was photographed on a UV light box.

### Two-dimensional nuclease activity assay

Protein extracts were resuspended in rehydration buffer (7 M urea, 2 M thiourea, 2% 3-[(3-cholamido propyl)-dimethylaminio]-1-propane sulfonate (CHAPS), 14 mM DTT, 18 mM Tris–HCl and 0.001% (w/v) bromophenol blue) and loaded onto 7 or 11 cm pH 4-7 linear IPG strips (Amersham Biosciences) for the first dimension. For nuclear extracts and dephosphorylated proteins rehydratation buffer were added by buffer exchange using Protein Desalting Spin Columns (Pierce) according to the manufacture’s method. The strips were rehydrated for 6 h at room temperature and isoelectric focusing (IEF) was performed in a IPGphor system (Amersham Biosciences) through 10 h at 50 V, 1.5 h at 500 V, 1.5 h at 1000 V, 1.5 h at 2000 v, 1.5 h at 4000 V, 2 h at 8000 V, and then holding at 8000 V until a total of at least 65000 Vh was reached. Afterwards, IPG strips were equilibrated for 15 min in equilibration buffer (50 mM Tris, pH 8.8, 6 M urea, 2% (w/v) SDS, 30% glycerol and 0.04% (w/v) bromophenol blue and then in equilibration buffer containing 10 mg^-1^ DTT for 15 min. Proteins were resolved in the second dimension using 10% SDS polyacrylamide gels and DNase activity assay were performed as we described above.

### Western blot analysis

The extracts were subjected to SDS/PAGE (10% acrylamide). After electrophoresis, the proteins were electroblotted onto nitrocellulose membranes (Amersham Bioscience) at 0.8 mA cm^-2^ for 1 h using the semi-dry transfer kit (Bio-Rad). The membranes were blocked with PBS-T buffer (20 mM Tris–HCl, pH 7.5, 150 mM NaCl, 0.1% Tween-20) containing 10% (w/v) powdered milk, and proteins were immunochemically labeled by overnight incubation of the membranes at 4°C in 20 ml of PBS-T and 1:1000 Histone H3 antibody (Cell Signaling Technology®) or 1:500 polyclonal GriP mutase [42]. Subsequent detection was performed by a chemoluminiscence assay (anti-rabbit IgG horseradish peroxidase conjugate from Roche) and SuperSignal® West Femto Maximum Sensitivity Substrate (Pearce).

### Mass spectrometry

Protein spots were excised from two-dimensional gels, trypsin digested and identified by ESI-Q-TOF-MS/MS. Proteins were identified either by peptide mass fingerprinting using matrix assisted laser desorption ionization-time of light mass spectrometry (MALDI-TOFMS) or by peptide sequencing. In the last case, the nanoliquid chromatography methodology coupled to nanoelectrospray tandem mass spectrometry (ESI-Q-TOF-MSMS) was carried out at the Proteomics Platform of the Barcelona Science Park. The software packages Protein Prospector v 3.4.1 (UCSF Mass Spectrometry Facility, University of California) and MASCOT were used to identify the proteins from the PMF data. The SEQUEST software (Thermo-Instruments, Spain) was used for preliminary protein identification from the tandem mass spectra analysis followed by manual sequence data confirmation. Sequence searching was performed on UniProtKB/Swiss-Prot protein knowledgebase and NCBInr databases 20050416 (2.440.549 sequences; 825.977.590 residues) and 20060729 (3.822.560 sequences; 1.317.468.070 residues) using protein full range of *Mr* and *pI*. No taxonomy restriction was applied. We used the following parameters for the searches: 1 missed cleavage; fixed and variable modifications were carbamidomethyl of cystein and oxidation of methionine, respectively. The peptide mass and fragment tolerance were 200 ppm and 0.25 Da, respectively. For MASCOT searching, individual ion scores >47 indicated identity or extensive homology (p < 0.05).

## Competing interests

The authors declare that they have no competing interest.

## Authors’ contributions

NSP and CV designed and performed most of the experiments, analyzed the data and wrote the manuscript. Both authors read and approved the manuscript.

## Supplementary Material

Additional file 1: Figure S1 Influence of IAA and DTT on the nuclease activity. Nuclease activity assays performed with 15 μg of total protein extracted from maize cultured cells grown in the presence of 0.5 μM CPT (+) or in control medium with DMSO (-) during 30 days. The assays were carried out in the presence of 1 mM CaCl_2_ and 1 mM MgCl_2_, 25 mg ml^-1^ iodoacetamide (IAA) and/or 10 mg ml^-1^ dithiothreitol (DTT) at pH 7.5. Click here for file

Additional file 2: Figure S2 Proteins associated with the nuclease activity in two-dimensional in-gel assay analyzed by MALDI-TOF MS. Identification of spots of nuclease activity in nuclease assays of nuclear protein extracts obtained from maize cultured cells after 30 days of incubation in the presence of 0.5 μM CPT. Click here for file

Additional file 3: Figure S3 Alignment of amino acid sequences (ClustalW) of type I endonucleases from Arabidopsis, nucleases PA3 from rice and the deduced protein sequences from maize mRNA. Conserved residues among all sequences are marked in grey and conserved residues of the tryptic peptide identified by mass spectrometry are marked in red. Maize protein identified by mass spectrometry sequences NP_001148452 (LOC1002820) and maize sequences (LOC100282067, NP_001123591, LOC100282147, LOC100272515 y LOC100273742). Rice sequences: OsENDO1 (LOC_Os01g03730), OsENDO2 (LOC_Os04g55850.1), OsENDO3 (LOC_Os01g03740), OsENDO4 (LOC_Os004g54390) and Arabidopsis sequences: AtENDO1 (At1g11190), AtENDO2 (At1g68290), AtENDO3 (At4g21590), AtENDO4 (At4g21585), AtENDO5 (At4g21600). Click here for file
