# Supplementary figures and images for: Identification of a type I Ca2+/Mg2+-dependent endonuclease induced in maize cells exposed to camptothecin
Source: BMC Plant Biol. 2013 Nov 20;13:186. doi: 10.1186/1471-2229-13-186 (PMC4225560; doi:10.1186/1471-2229-13-186)

## Slide 1
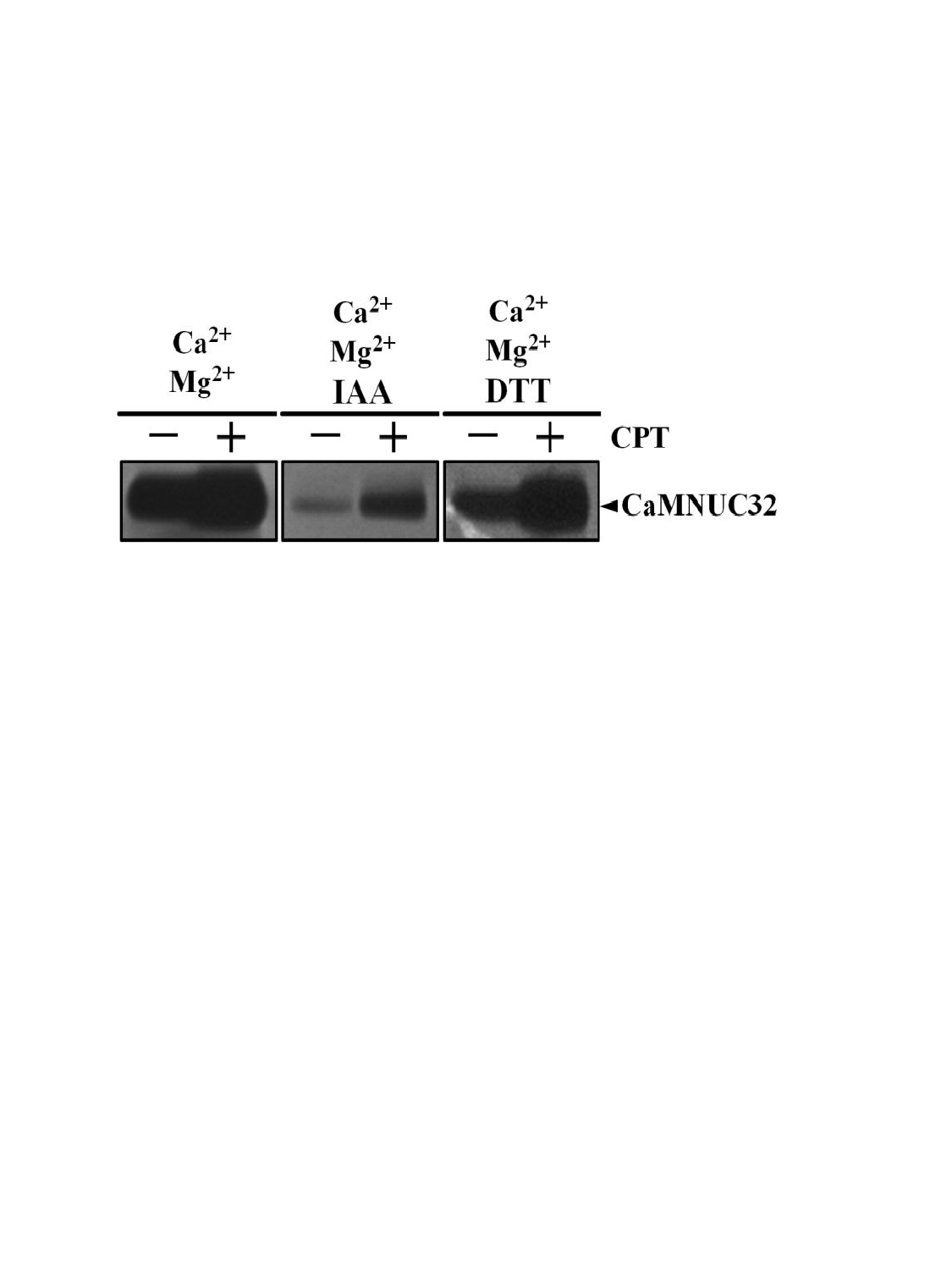

Supplement: Additional file 1: Figure S1 — Influence of IAA and DTT on the nuclease activity. Nuclease activity assays performed with 15 μg of total protein extracted from maize cultured cells grown in the presence of 0.5 μM CPT (+) or in control medium with DMSO (-) during 30 days. The assays were carried out in the presence of 1 mM CaCl2 and 1 mM MgCl2, 25 mg ml-1 iodoacetamide (IAA) and/or 10 mg ml-1 dithiothreitol (DTT) at pH 7.5. [file 1471-2229-13-186-S1.pptx]

## Slide 1
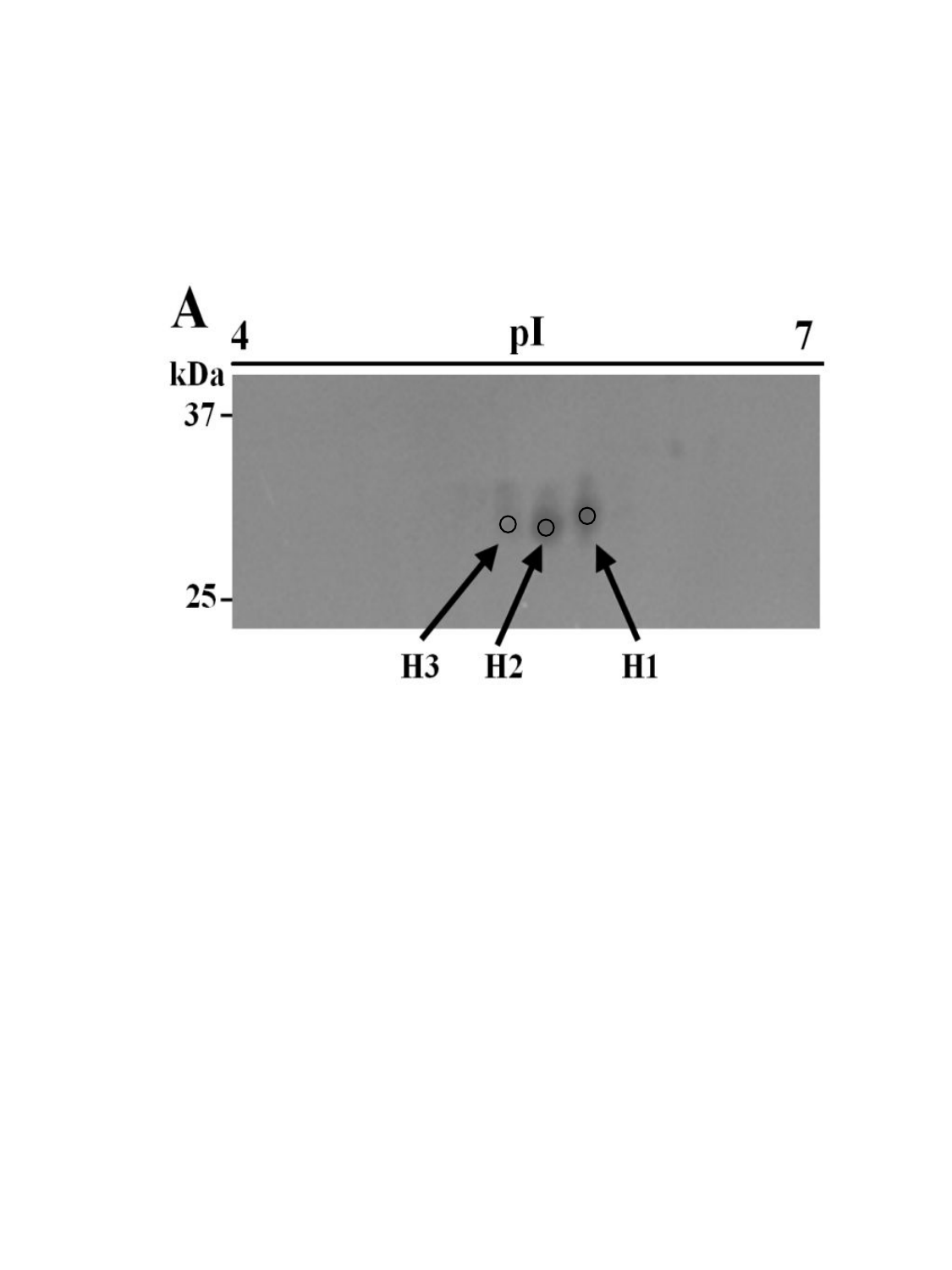

Supplement: Additional file 2: Figure S2 — Proteins associated with the nuclease activity in two-dimensional in-gel assay analyzed by MALDI-TOF MS. Identification of spots of nuclease activity in nuclease assays of nuclear protein extracts obtained from maize cultured cells after 30 days of incubation in the presence of 0.5 μM CPT. [file 1471-2229-13-186-S2.pptx]

## Slide 1
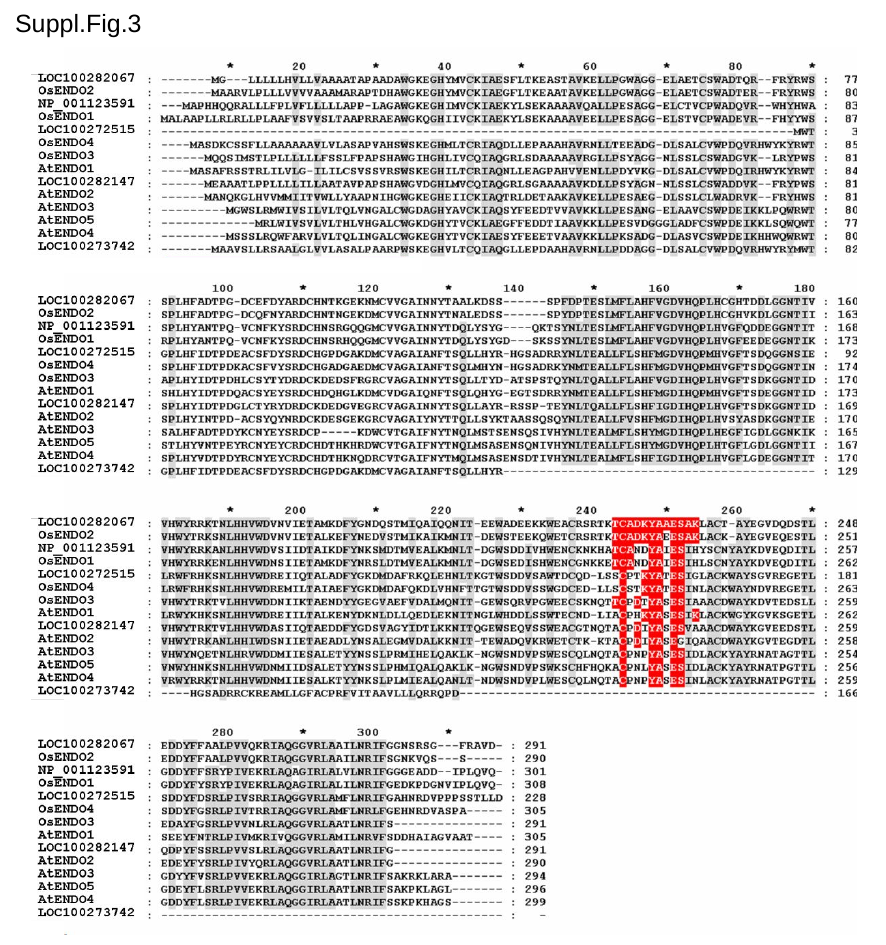

Suppl.Fig.3

Supplement: Additional file 3: Figure S3 — Alignment of amino acid sequences (ClustalW) of type I endonucleases from Arabidopsis, nucleases PA3 from rice and the deduced protein sequences from maize mRNA. Conserved residues among all sequences are marked in grey and conserved residues of the tryptic peptide identified by mass spectrometry are marked in red. Maize protein identified by mass spectrometry sequences NP_001148452 (LOC1002820) and maize sequences (LOC100282067, NP_001123591, LOC100282147, LOC100272515 y LOC100273742). Rice sequences: OsENDO1 (LOC_Os01g03730), OsENDO2 (LOC_Os04g55850.1), OsENDO3 (LOC_Os01g03740), OsENDO4 (LOC_Os004g54390) and Arabidopsis sequences: AtENDO1 (At1g11190), AtENDO2 (At1g68290), AtENDO3 (At4g21590), AtENDO4 (At4g21585), AtENDO5 (At4g21600). [file 1471-2229-13-186-S3.ppt]
